# Is “Wild” a Food Quality Attribute? Heavy Metal Content in Wild and Cultivated Sea Buckthorn and Consumers’ Risk Perception

**DOI:** 10.3390/ijerph18189463

**Published:** 2021-09-08

**Authors:** Ruxandra Malina Petrescu-Mag, Iris Vermeir, Carmen Roba, Dacinia Crina Petrescu, Nicoleta Bican-Brisan, Ildiko Melinda Martonos

**Affiliations:** 1 Faculty of Environmental Science and Engineering, Babes-Bolyai University, 30 Fantanele Street, 400294 Cluj-Napoca, Romania; malina.petrescu@ubbcluj.ro (R.M.P.-M.); carmen.roba@ubbcluj.ro (C.R.); nicoleta.brisan@ubbcluj.ro (N.B.-B.); ildiko.martonos@ubbcluj.ro (I.M.M.); 2 Gembloux Agro-Bio Tech, University of Liège, 2 Passage des Déportés, 5030 Gembloux, Belgium; 3 Department of Marketing, Innovation and Organization, Faculty of Economics and Business Administration, Ghent University, 9000 Ghent, Belgium; iris.vermeir@ugent.be; 4 BE4LIFE, Research Center on Sustainable, Healthy and Ethical Consumption, Ghent University, 9000 Ghent, Belgium; 5 Faculty of Business, Babes-Bolyai University, 7 Horea Street, 400174 Cluj-Napoca, Romania

**Keywords:** sea buckthorn, mining area, contamination, consumption, health, herbal supplements

## Abstract

Globally, the consumption of herbal supplements is on an upward trend. As the food supplement industry thrives, so does the need for consumers’ awareness of health risks. This contribution is grounded on two assumptions. Firstly, not always “wild” is a food quality attribute, and secondly, the food chain is judged as a noteworthy route for human exposure to soil contamination. Sea buckthorn (SBT) was selected for investigation due to its versatility. In addition to its wide therapeutic uses, it is present in ecological rehabilitation which may raise concerns regarding its safety for human consumption as a consequence of the accumulation of contaminants in the plant. The study aims to discover if the objective contamination of SBT with toxic residues is congruent with people’s subjective evaluation of SBT consumption risk. A quantitative determination of heavy metals was performed by atomic absorption spectrometry. The metals abundance followed the sequence Fe > Cu > Zn > Mn > Cr > Ni > Pb > Cd. Quantitative data on consumers’ subjective risk evaluations were collected through an online survey on 408 Romanians. Binary logistic shows that the consumption of SBT is predicted by the perceived effect of SBT consumption on respondents’ health. The study confirms that the objective contamination of wild and cultivated SBT is in line with the perceived contamination risk. It is inferred that a joint effort of marketers, media, physicians, and pharmacists is needed to inform consumers about the risks and benefits of SBT consumption.

## 1. Introduction

Emerging evidence of health-related benefits of plant-derived supplements raises consumers’ interests, and their consumption has taken over other products of animal origin [1]. As the food supplement industry thrives, so does the need for increased consumer awareness of the safety of these products. The lack of information on raw materials’ traceability, missing or inadequate labeling can all compromise the food supplements safety. Consumers’ food safety risk information-seeking behavior can significantly contribute to improve safety awareness and prevent food safety risks [2]. The available evidence shows that at a global level, herbal medicinal products, supplements or nutraceuticals consumption is on an upward trend, with over 80% of people worldwide using them as part of primary healthcare [3].

Italy is the first in terms of the dietary supplements market in the European region with a value of EUR 1.6 billion in 2020, while Romania is ranked 15th (with EUR 101 million, an increase of 71% since 2015) [4]. Nevertheless, official statistics on plant trade and consumption are scant and not very informative as often they are part of the informal economy [5].

There is a wide range of terms used for natural-based products aiming to depict their role in enhancing consumers’ healthiness. There are inconsistencies and contradictions in the definitions of “natural products”, “nutraceuticals”, “functional foods”, “herbal foods” terms which show uncertainty about what they really are [6]. Aronson [6] is one of the many authors who blame the lack of agreed definitions of these terms, which he considers “unhelpful” and should be abandoned in favor of more precise terminology. That is why, for the present study, the term “SBT” was preferred. The sea buckthorn products (*Hippophae rhamnoides* L., Elagnaceae, abbreviated hereafter as SBT) are among the most consumed and sought after by Romanian consumers.

The SBT is a shrub naturally distributed in Asia and Europe [7], known for its benefits both as food and as medicine. Scientific literature reports the SBT healing properties, highlighting its content of minerals, vitamins, and other bioactive substances [8,9,10,11]. Positive effects of SBT were reported based on clinical investigations and animal experiments concerning high cholesterol, anticancer effects [12,13,14], immunity [15], cardiovascular diseases [11,16], and irradiation dermatitis [17]. Practically, SBT has become so popular for its therapeutic and cosmetic purposes that hundreds of SBT products are available in the market [16]. However, the quality of SBT plants is crucial as the active substance concentration can vary, depending on environmental and other factors (e.g., how the berries are cultivated and harvested). They all together determine the efficiency and safety of the SBT products.

SBT products could be regarded as safe products due to consumers’ misconception of valuing “natural products” as “safe” since they are derived from a “natural” source [3]. However, the food chain (soil–plant–human) is a significant pathway for human exposure to soil contamination [18]. Thus, SBT products could contain heavy metals when they are made of SBT grown on contaminated soil. Scientific literature testifies that heavy metal pollution of mined areas can cause health problems due to excessive dietary accumulation of heavy metals in the human body [19,20,21]. In two Romanian localities (Copsa Mica and Baia Mare), Lacatusu et al. [22] found that pollution with Pb and Cd in soil and vegetables may reduce the average age at death by 9–10 years within the polluted area. Kumar et al. [23] warn that special attention should be paid to Pb since between 20% and 70% of ingested Pb is absorbed by the human body.

Consequently, this study analyzes the heavy metal concentration of SBT from different locations (tailings dumps, farmers’ gardens, and supermarket). In addition, Romanian consumers’ perceptions of risks associated with various hazards related to SBT products are investigated. A particular focus is placed on the perceived risk of the SBT content of toxic residues due to the presence of SBT on contaminated land from mining activities. The present study adopts the understanding of Schroeder et al. [24] on risk perception, seen as consumers’ views about the risk inherent in a particular situation. More precisely, perceptions of SBT product safety risk refer to what consumers believe is the amount of health risk they face from consuming SBT products.

The investigation of consumers’ perception of different risks associated with SBT is a topical issue in consumer studies. Therefore, the study aims to discover if the objective contamination of SBT with toxic residues is congruent with people’s subjective evaluation of SBT consumption risk. Several objectives were set to achieve this aim. Firstly, to make a comparative assessment between the quantity of metals in SBT from three location types—tailings dumps, farmers’ gardens, and supermarket. Secondly, to reveal SBT consumption characteristics (SBT average consumption, SBT consumption deterrents, preferred type of SBT, importance of SBT wild origin.). Thirdly, to identify people’s perceptions regarding the risk associated with SBT consumption. Finally, to discover if SBT health benefits and risk perception can predict SBT consumption.

The present contribution adds to the consumer literature by enhancing the understanding of Romanian consumers’ perception of SBT products within the European geographical space. According to Schulp et al. [25], around 14% of all EU citizens collect wild food occasionally. Moreover, the study provides a context for observing if the actual and objective contamination of wild and cultivated SBT is in line with the risk of contamination perceived by people. To the extent of our knowledge, this is the first study dedicated to Romanian consumers of SBT. The paper offers detailed information about SBT consumption preferences, such as consumption frequency, preference for wild versus cultivated SBT. It also discloses Romanians’ risk perception associated with SBT consumption, being the first contribution that investigates many possible risks that may raise consumers’ concerns.

## 2. Theoretical Framework and Hypotheses

A recent report [26] showed that, at the EU level, potentially polluting activities took or are still taking place on about 2.8 million sites. In Romania, there are 210 sites where potentially polluting activities are taking place [27]. Mining and metallurgy are activities that have been carried out in Romania for more than 2000 years. One of the areas that raises the most significant environmental problems is the Jiu Valley Carboniferous Basin (in the south, south-west of Romania, which was one of the sample locations in the present study), where the distribution of coal reserves is concentrated. According to Faur et al. [28], in the Jiu Valley, the total area occupied by waste dumps is over 270 ha, and the volume of waste material stored is about 40 million m^3^. Heavy metals and acid leaks transformed the mining dumps into environmental risks [29]. Soil reclamation works are needed, and one of the most popular plant species used is SBT [together, for example, with the Spruce (*Picea abies*), acacia (*Robinia pseudacacia*), willow (*Salix babylonica*)] [30].

SBT is a versatile plant. In addition to its wide therapeutic and cosmetic uses, it is also present in ecological rehabilitation, which may raise concerns regarding its safety for human consumption. Zhao et al. [31] showed that SBT was one of the best plant species for activating soil development in the early reclamation stage [31]. In Romania, SBT is largely used for ecological restoration. It is a nitrogen-fixing species, and it can grow on marginal land, which recommends it as an excellent plant for soil improvement [32]. Small et al. [33] also reported the suitability of SBT for improving soil’s physical characteristics and fertility. Within this context, due to the high adaptability of SBT to contaminated soils and the growing SBT’s industrial utilization, the cultivation of SBT has seen a rapid increase in Romania during the past five years.

Consequently, special consideration should be put on SBT heavy metal accumulation. Eeva et al. [34], Ettler et al. [35] and Salemaa et al. [36] are among the many authors who observed that dwarf shrubs (such as SBT) were prone to absorb metals from the soil due to their shallow roots that favored the absorption of nutrients mainly from the upper soil layers where airborne metals accumulated. The following hypothesis is formulated:

**Hypothesis** **1** **(H1).** *The SBT from spontaneous (wild) flora contains more toxic residues compared to cultivated SBT*.

While scientific evidence exists to support the use of a wide variety of herbs for some health problems, there is still a great concern about the origin of the herbs available in the marketplace. Previous studies found that many people believe that herbal products are safe since they are natural [37]. Still, few studies assess perceptions and beliefs regarding herbal products [38]. However, the literature reports also safety concerns about natural products. They range from the lack of standardization of natural products, which causes variations in herbal content [39], misidentification of herbs [40], contamination during manufacturing or even the presence of undeclared stimulants [41], to herb-drug interactions [16]. While a large amount of research has explored consumer risk perception of particular hazards related to food, such as GMOs [42,43], additives [44,45] or pesticides [46], no study to date has investigated consumers’ risk perception for SBT products, to the best of our knowledge. Due to the national specific context (mentioned at the beginning of this section), particular attention was put on the perceived risk of the SBT content of toxic residues since they often grow on contaminated land from mining activities. We propose the following hypotheses:

**Hypothesis** **2** **(H2).** *People perceive a higher risk of contamination for wild SBT compared to cultivated SBT*.

**Hypothesis** **3** **(H3).** *There is a difference among people who prefer SBT from wild sources, those who prefer cultivated, and those with no preference regarding the perceived risk of wild (H3a) and cultivated (H3b) SBT contamination with toxic residues since they grow on contaminated land*.

Furthermore, in a worldwide context where healthy eating is a target of international and national health strategies, health benefits and safety are the main themes of factors influencing consumer’s decision on nutraceuticals intake, but it has not been established whether health and perceived risk determine the consumption of SBT products. Health is one of the main factors that influence consumers’ food choices [47,48,49] and it includes two aspects (similarly to the consequence of any action—good or bad). The positive one refers to the benefits that people pursue to obtain by consuming a product and the negative one concerns health damages caused by the consumption of a specific food. These two effects on health were included in the present analysis by asking about the SBT effect of health, in general, with answer options covering both types of effects (thus, referring to benefits and also damages). Moreover, specifically related to study focus, these effects were investigated by asking about the SBT contamination risk and other risks (which relate to the negative effect of consuming a certain food product). We propose that:

**Hypothesis** **4** **(H4).** *SBT consumption is predicted by (i) the perceived risk of contamination with toxic residues of wild SBT, (ii) the perceived risk of contamination with toxic residues of cultivated SBT, (iii) the average level of concern about various risks related to SBT, and (iv) the perceived effect of SBT consumption on respondent’s health*.

## 3. Materials and Methods

**Figure 1:**
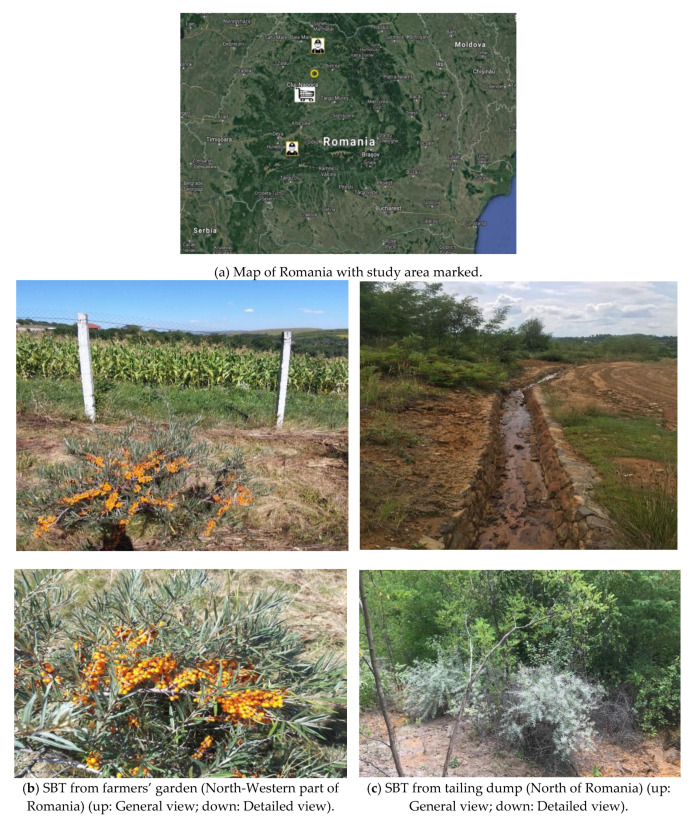
Study area and sample collection. Source: (**a**) Adapted from [50]; (**b**,**c**) photos from the authors’ archive. Legend: 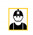 Represents the tailings dumps where samples 1 to 4 were sampled; the farmers’ garden location (for samples 5 and 6) is represented in the yellow circle; 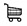 Indicates the location of the supermarket (sample 7).

SBT berry samples were hand-harvested in July 2020 from both non-polluted areas [two samples from two farmers’ gardens (Figure 1), Cluj County, Corpadea village], four samples from polluted areas [tailings dumps from coal and non-ferrous mining Figure 1, and one was from fruits bought from a supermarket (Figure 1)]. Spontaneous and cultivated SBT can both grow on contaminated and clean soil.

The sampling points from polluted areas were selected to reflect the possible impact of heavy metal pollution sources (emission and dispersion of pollutants from mining activities). As highlighted in the Theoretical Framework and Hypotheses section, quality is crucial in differentiating supermarkets’ products from those of traditional markets [51] and even from local shops. Therefore, it was considered relevant to have one sample from SBT purchased from a supermarket. However, it should be mentioned that this product was from a Romanian producer, and it was the only brand available at that moment. At the same time, many consumers do not trust large-scale food production, processing, and distribution systems. They are looking for an alternative for restoring local food markets [52,53] and, thus, contributing local economy and community support. When more and more people are interested in local products, it is relevant to sample berries from local farmers who cultivate and trade SBT.

After sampling, the fruits were rinsed with ultra-pure water, dried at 105 °C for 24 h, grounded, homogenized, weighed (0.4 g), and then the acidic microwave digestion was performed (Speedwave Berghof system; Analytik Jena, Jena city, Germany), using 5 mL of HNO_3_ (65%) and 3 mL of H_2_O_2_ (30%). The obtained clear solutions were then brought to a constant volume (25 mL), with HNO_3_ (0.2%) and filtered (0.45 µm). The fruits purchased from the supermarket were frozen in the laboratory, they were thawed, rinsed with ultra-pure water, dried, and processed according to the protocol previously discussed. The quantitative determination of metals (Cu, Zn, Fe, Cr, Cd, Pb, Ni, Mn) was performed by atomic absorption spectrometry (AAS), using a ZeeNIT 700 system (Analytik Jena; Jena city, Germany) equipped with a single-element hollow cathode lamp, an air-acetylene burner, and a graphite furnace. The operating conditions were met according to the manufacturer requirements mentioned in the user manual. The external standard method was used to quantify the analytes. The calibration curves were plotted using standard solutions prepared by serial dilutions of the standard stock solution (1000 mg/L in 1% *w*/*w* HNO_3_) (Merck). The limit of detection (LOD) ranged between 0.08 µg/L (Cd) and 0.66 µg/L (Ni) for the electrothermal atomization in the graphite furnace.

Quantitative consumer data were collected through a survey conducted online. A sample of 408 Romanian consumers was selected, and data were collected by a specialized company. The sample was representative at the country level by gender, age, and geographical distribution (considering the nine development regions of Romania). The investigated variables, the questions, and answer options are presented in Table A1 (Appendix A). Data were analyzed in Excel and SPSS (Statistical Package for the Social Science; software source: Ghent University, Ghent, Belgium). Univariate analyses, Wilcoxon test, Kruskal Wallis test, and binary logistic regression were run to fulfill the research objectives.

## 4. Results

The sample characteristics are presented in Table 1.

Frequency data from Table 2 show that the overwhelming majority of interviewed people consume SBT. The main obstacle in consuming SBT is taste. At the same time, respondents placed “pesticides”, “unknown origin”, and “lack of hygiene standards” in the top three in terms of their concerns about various risks related to SBT.

The content of metals of SBT berries was analyzed to test whether the SBT from spontaneous flora consists of more toxic residues compared to those from cultivated plants (see Table 3). Significant differences in the element content were found, depending on the sampling place. The samples collected from the tailings dumps areas (spontaneous flora) had a considerably higher level of metals than those sampled from non-polluted areas (cultivated plants). The highest content of copper and zinc was registered in sample 2, which can be related to the location of the sample on the tailings dumps, because high values of copper and zinc can be associated with the mining activities. The metals abundance follows the sequence Fe > Cu > Zn > Mn > Cr > Ni > Pb > Cd. The analyses were performed in triplicates and the relative standard deviations (RSD) for the individual samples showed no significant differences. The RSD were up to 6.8% (Cd and Pb), 6.5% (Cr), 4.8% (Ni), 2.5% (Cu), 1.6% (Zn) 1.2% (Mn), and 1.1% (Fe).

While literature reports [55,56,57] different national threshold values for some of the heavy metals found in vegetables, to the authors’ knowledge, there is no paper in the scientific literature in the English language dedicated to berries and other small fruits, in general, and SBT, in particular. Consequently, as a reference value for Pb and Cd, the Commission Regulation (EC) No. 1881/2006 of 19 December 2006 and Commission Regulation (EU) 2015/1005 amending Regulation (EC) No. 1881/2006 was consulted in regards to the maximum levels of lead in certain foodstuff. For Pb in berries and other small fruits, the General standards for contaminants and toxins in food and feed (CODEX STAN 193-1995) [54] indicated the recommended value of 0.1 mg/kg. The content of Pb and Cd in the SBT berries was within the permissible limit, according to the EU regulation (0.2 and 0.05 mg/kg f.w., respectively). The result indicates that they do not represent a risk factor for the consumer’s health. The level of Cd in some SBT berries sampled from the tailings dumps is close to the safe limits, requiring careful monitoring of cadmium in fruits collected from those locations or similar ones.

In Romania, maximum limits for arsenic and heavy metals in food were regulated in the Romanian legislation within the Hygienic-sanitary norms for food (published in 1999), which was repealed in 2009. Consequently, there is a legislative vacuum regarding all these limits.

A binary logistic regression analysis was used to test whether SBT consumption is predicted by the perceived risk of contamination, concern about various risks related to SBT, and perceived health effects of consuming SBT (H4). The Omnibus Tests of Model Coefficients indicates how well the model performs, and it generated a highly significant value (*p* < 0.005) and a chi-square value of 57.133 with 4 degrees-of-freedom. The *p*-value of the Hosmer and Lemeshow Test is greater than 0.05 (*p* = 0.605) signifying a good fit and support of the model. Between 13.2% and 20.1% of the variability in consuming SBT is explained by the perceived SBT effect on the respondent’s health, based on the Cox and Snell R Square and the Nagelkerke R Square values. People who perceive the SBT consumption effect as beneficial are more likely to consume SBT. The perceived risks and the concerns related to various risks do not have predictive power (Table 4).

The Wilcoxon test indicated that the perceived risk of contamination with toxic residues of SBT was significantly higher for wild SBT than cultivated SBT (Z = −2.336, *p* = 0.020), confirming the second hypothesis (H2).

The Kruskal Wallis test with the post-hoc test and Bonferroni correction were run to test H3 (a,b) (Table 5). Firstly, the Kruskal Wallis test showed a statistically significant difference among some of the three groups regarding the perceived risk of contamination with toxic residues of wild SBT (*p* = 0.045) (but this test does not indicate where the differences are). Then, the post-hoc test with Bonferroni correction were run and indicated that people who preferred wild SBT perceived a lower risk of wild SBT compared to people who preferred cultivated SBT and to people with no preference.

In the case of perceived risks of cultivated SBT, the Kruskal Wallis test indicated that there was no significant difference between any of the three groups (*p* = 0.919).

A significant difference in the perception of contamination risk for wild SBT was found between different genders, with women perceiving higher risk (U = 17341.5, Z = −2.983, *p* = 0.003). No gender differences were found for the perception of contamination risk in the case of cultivated SBT (U = 18828, Z = −1.680, *p* = 0.093). A Spearman’s rank-order correlation indicated a weak and negative correlation between age and the perception of contamination risk both for wild (r_s_ = −0.144, *p* < 0.005) and cultivated SBT (r_s_ = −0.158, *p* < 0.005). No differences in perceived risk of contamination neither for wild SBT nor for the cultivated ones were found for different living environments, education levels, and income.

The overall situation concerning the acceptance and rejection of the proposed research hypotheses is presented in Table 6.

## 5. Discussion

The quantitative determination of Cd and Pb performed by atomic absorption spectrometry indicated, for all the samples, values within the threshold limits of the EU regulations [58,59]. All the values for the investigated heavy metals (mg/kg fresh weight) concentration in the SBT berries were much lower in specimens from cultivated plants than from spontaneous flora, thereby confirming hypothesis 1.

The Cd, Pb, and other metals’ concentrations were the lowest in the cultivated SBT samples (5, 6, 7), except for Ni, compared to the wild flora. Based on an assessment of 18 environmental metals in subsistence species sampled in a Russian region, Dudarev et al. [60] found exceedances of Cd and Cr in wild berries. Therefore, metal accumulation in both SBT cultivated and spontaneous flora should be considered and monitored during the selection and processing of these fruits.

For the rest of the heavy metals (Cu, Zn, Fe, Cr, Ni, Mn), further assumptions may open a broad array of discussions as there are no mandatory or recommended maximum levels. The laboratory analyses indicated the highest values on tailings dumps for Fe, Cu, and Zn. Gutzeit et al. [61] found a content of 0.616 mg/kg of Ni, 1.779 mg/kg of Zn, and 0.988 mg/kg of Cu in SBT from a commercial planting in Romania. If we compare those results with the ones obtained in the present research, it is observed that for Ni and Zn, our values are lower for cultivated berries, while for Cu are higher. These variations in heavy metal content are due both to the soil natural element composition and the maturity level of the berries [61]. Thus, a general conclusion cannot be drawn if we refer to plants from contaminated sites. Therefore, permanent monitoring of heavy metal presence in food is required as various health disorders are reported due to chronic exposure [19,62].

From the frequency data, it was observed that 77.2% of the respondents consumed SBT (Table 2). Data show that Romanian consumers tend to prefer wild SBT to the cultivated one and the importance of being of wild origin is highly relevant to them. The healthy bioactive compounds are probably those which lean consumers’ preference towards wild SBT. Di Vittori et al. [63] underline the higher level of nutritional attributes of wild berries when compared to the cultivated ones. Similar to this finding, other research reports the perceived health properties of wild food plants [64]. As posited in several studies [65,66], consumers perceive wild food as excellent product quality, with positive health effect, and as exclusive products compared to mass ones. For example, the primary motivation of West Sumatra farmers to consume wild plant foods was based on their perceived attribute of “unpolluted” natural foods [67]. Several sources point out the association between wild fish and naturalness compared to aquaculture. Thus, in a study by Verbeke et al. [68], consumers perceived wild fish as more natural and healthy. Consistent with previous research, the findings of the present paper reveal that in balancing exposure to possible toxic residues of wild SBT and the potential health benefits, the benefits outweigh the risks.

From a practical perspective, these data can inform managers that Romania is an important market for SBT consumption. They should focus more on how to communicate the different risks that consumers associate with SBT consumption. There is a solid need to remove their concern for risks such as pesticides or heavy metals. The label “Made of wild SBT”, together with information about the traceability of ingredients or care for hygiene standards, could bring a competitive advantage.

The present findings illustrated that wild SBT was perceived as more harmful than the cultivated one, probably due to the spread of SBT on contaminated sites. However, it should be underlined that this perception did not influence the preference for wild SBT. This finding confirms Hartmann et al.’s [69] and Green et al.’s [70] results research. They postulated that the risks associated with food consumption are not the most relevant factors in food consumption decision-making. In line with their findings, no difference was found between consumers and non-consumers of SBT regarding the perceived risk of contamination of SBT due to their large spread on polluted land (neither for wild SBT nor cultivated SBT). Furthermore, the perceived effect of SBT consumption on the respondent’s health has a good prediction capacity of the consumption of SBT (Table 4). However, the perceived risk of contamination with toxic residues since they grow on contaminated land for wild and cultivated SBT, respectively, and the average concern about various risks related to SBT risk consumption do not contribute to consumption prediction.

The perceived risk of contamination with toxic residues of cultivated SBT was similar for people who preferred SBT from wild plants, those who preferred cultivated SBT, and those who were indifferent. This means that the strategies for promoting cultivated SBT to these three groups can be similar in relation to the mention of the contamination risk with toxic residues of cultivated SBT.

When considering the risk perception of wild and cultivated SBT, it must be underlined that SBT-based products should not be regarded as a regular food product, but as a dietary supplement, as a food-medicine. It is worthwhile to note that SBT intake is based on its possible health improvement characteristics. Thus, consumers should pay greater attention to the risks associated with SBT consumption. Due to the spread of SBT on contaminated sites, SBT is prone to heavy metal contamination, as explained in the previous sections. Therefore, the consumption of this plant requires caution, even if, in the present study, the values of heavy metals were within the legal thresholds. A study of Saini et al. [71] that investigated the capacity of *Hippophae* leaf extract concentration to regulate the antioxidant and prooxidant effects on DNA, suggested that dietary supplements prepared from *Hippophae* should have low metal content. Equally important is to point out that it cannot be concluded that risk perception related to a particular product, such as SBT, influences general consumer trust in food safety [72].

Several studies highlight that consumers with different socio-demographic characteristics perceive food risks differently [73,74]. The present contribution indicated that there is a statistically significant difference according to gender in the perception of contamination risk for wild SBT. Women perceived higher risks than men, a result in line with other findings reported in the risk food literature [75] that is often explained through “social roles and gender roles” [76].

From a practical perspective, managers could target consumers less likely to purchase SBT products. They could focus on taste as it was indicated as the main deterrent in consuming SBT. Different product combinations with apples, honey, juices, and marmalade can create a competitive advantage. SBT availability was in the second place as an obstacle to SBT consumption. Thus, authorized shops (e.g., supermarkets and pharmacies) could offer SBT products on their shelves. The findings suggest that Romanian consumers perceive higher values of wild SBT. In this way, marketers should allocate their resources to promote SBT products from spontaneous flora while, of course, endorsing the message of their safe consumption.

There are several limitations of this study that the authors acknowledge. The AAS system used for the present laboratory analyses limited the number of investigated metals to the number of existing hollow cathode lamps. Then, this empirical evidence on consumers’ perceptions of SBT-based products is limited to a geographical market. Consequently, the findings are context-dependent and cannot be extrapolated to other markets and consumers. The sample comprised adult participants mainly from urban areas (84.1%). Thus, the results could be generalized only to this population. Further research should be conducted to reveal other factors that can influence consumers’ risk perception, as it is known that perception of food risks is multifactorial [69]. Additionally, little is known about the public’s attitudes and beliefs regarding herbal medicines in general, and SBT products, in particular. Since previous consumers’ experience influences repurchasing decisions and how they recommend the product to others [77], future studies should focus more on this market segment to reveal additional aspects about risk perceptions, willingness to buy SBT products, willingness to pay for wild SBT, SBT consumption frequency or perceived efficacy. The aim of this study was to compare the SBT objective contamination (determined through laboratory tests) with the subjective one (the one perceived by people) and not to estimate the SBT intake (which would have required a different questionnaire). Currently, there are no available national statistics on annual sea buckthorn consumption. For these reasons, it was not possible to calculate here the ingestion dose of heavy metals via sea buckthorn consumption, and consequently, the food contaminant exposure assessment, which suggests to investigate this direction in future research.

## 6. Conclusions

The present contribution focuses on a topical issue of the food supplements industry, that of consumers’ risk perception of herbal products, more precisely, SBT products. It is argued that the long-term accumulation of heavy metals in soil is one of the primary sources of food contamination in general, and SBT, in particular. SBT products are prone to toxic residue accumulation due to the Romanian-specific context of contaminated land from the mining legacy of the former communist regime. The quantitative determination of metals (Cu, Zn, Fe, Cr, Cd, Pb, Ni, Mn) performed by atomic absorption spectrometry indicated that all values fall within the legal limits (where they exist). However, differences in the element content were found, depending on the sampling place. SBT sampled from the tailings dumps areas had a considerably higher level of metals than those sampled from non-polluted sites. Currently, only the concentration of Pb and Cd is regulated. Thus, threshold values must be immediately endorsed under the national specific legislation. Moreover, regulation is required for berries such as SBT since it is evident that toxic accumulations differ between small and big fruits. This legal requirement is all more critical since SBT is one of the most consumed berries in Romania [78].

The statistical analyses indicate that for Romanian consumers, the perceived health benefits of SBT outweigh the perceived risks. The perceived effect of SBT consumption on the respondent’s health predicts the consumption of SBT, while the perceived risk of contamination with toxic residues since they grow on contaminated land for wild and cultivated SBT, respectively, and the average concern about various risks related to SBT consumption, do not contribute to consumption prediction.

Finally, the study confirms that the objective contamination of wild and cultivated SBT is in line with the risk of contamination perceived by people. More precisely, the contamination revealed by laboratory tests is higher for wild SBT than cultivated SBT and people perceive higher risk for wild SBT, too. Furthermore, despite the high health benefits Romanian consumers associate with SBT (health benefit average value was 8.5 points out of 10), safety concerns still exist. These risk concerns put a great responsibility on producers and handlers to ensure product safety and consumers’ trust. Therefore, effective food risk communication strategies tailored to answer consumers’ risk perceptions can create a competitive advantage. With the rising use of SBT products, a multi-sectoral collaboration between marketers, mass media, physicians, and pharmacists is needed to inform and educate consumers about the risks and benefits of SBT consumption.

## Figures and Tables

**Table 1:** Socio-demographic profile of the sample (n = 408).

Variable	Frequency	Mean	Standard Deviation
**Gender**			
M	48.8%		
F	51.2%		
**Age** (years)		45.6	15.6
**Living environment**			
Urban	84.1%		
Rural	15.9%		
**Education**			
8 years	3.4%		
12 years	34.1%		
Graduate	62.5%		
**Income**			
Max 400/month	25.5%		
401–800 euro/month	45.6%		
801–1200 euro/month	11.3%		
1201–1600 euro/month	4.4%		
1601–2000 euro/month	2%		
Over 2000 euro/month	0.5%		
**No answer**	10.7%		

**Table 2:** Consumption preferences and perceived risks related to SBT.

Variable	Frequency	Average Value
**SBT products consumption ^a^**		
Yes	77.2%	
No	22.8%	
**Average no. of days with consumption ^b^**		78.9
**Reasons for not consuming SBT (frequencies are calculated for non-consumers) ^c^**		
I cannot find SBT	15.1%	
I do not have time	10.8%	
I do not care	14.0%	
I don’t like the taste	26.9%	
I don’t like the smell	7.5%	
SBT does not do me any good (I am allergic to it, it causes me acidity, etc.)	8.6%	
It seems complicated to me to consume it	6.5%	
I think it doesn’t have a significant beneficial effect on my health	7.5%	
I do not trust the quality of SBT available on the market	6.5%	
Another reason (I do not know its properties; my doctor did not recommend it; I use other plants)	11.8%	
**Preferred type of SBT ^a^**		
Wild	39%	
Cultivated	33.8%	
Indifferent	27.2%	
**Importance that the SBT are of wild origin ^a^**		82.3 (points)
**Risk of contamination with toxic residues of SBT because they grow on contaminated land ^a^**		
Wild		40.14%
Cultivated		36.34%
**SBT effects on respondent’s health ^a^**		8.5 (points)
**Concerns about various risks related to SBT ^a,d^**		
1. They are falsified	31.6%	
2. They are of low quality	41.2%	
3. They do not comply with hygiene standards	49.5%	
4. They contain pesticides	52.2%	
5. They contain hormones	29.4%	
6. They contain drugs residues	34.6%	
7. They contain additives	45.1%	
8. They contain GMOs	36.8%	
9. They contain toxic residues because they grew on lands contaminated with mining pollution	49.5%	
10. Their origin is unknown	50.7%	
11. They contain other ingredients which effect I do not know, and it can affect my health	46.3%	
12. They can have harmful effects when they are taken together with some drugs	44.9%	
13. They do not have the healing effect I want	32.8%	
14. They can create addiction	12.5%	
15. They were not verified/tested by the production company to see if they are safe for consumer health (e.g., for toxic substances)	43.6%	
Average risk ^e^		1.6 (points)

^a^ Results calculated for the entire sample (SBT consumers and non-SBT consumers); ^b^ results calculated only for SBT consumers (315 persons); ^c^ results calculated only for non-SBT consumers (93 persons); ^d^ results reflected the percentage of people who are concerned with the risk; ^e^ the average was calculated by summing up the answer values (1 or 2) and dividing the result to 408.

**Table 3:** Metal content (mg/kg fresh weight) in sea buckthorn berries.

Sampling Location	Fe	Cu	Cr	Pb	Cd	Zn	Ni	Mn
	(mg/kg Fresh Weight)							
Tailing dump (Lupeni coal mining —Hunedoara County), sample 1	19.37	7.38	0.59	0.033	0.032	8.54	0.56	4.21
Tailing dump (Lupeni coal mining—Hunedoara County), sample 2	19.30	66.44	0.80	0.053	0.041	54.13	1.12	3.95
Tailing dump (Vulcan coal mining —Hunedoara County), sample 3	25.89	3.56	0.88	0.047	0.039	6.28	0.16	1.92
Tailing dump (Plopiș-Răchițele non-ferrous mining—Maramureș County), sample 4	6.51	1.58	0.84	0.034	0.010	3.72	0.18	4.28
Farmer garden, sample 1 (Cluj county, Apahida, Corpadea village), sample 5	3.41	1.17	0.21	0.006	0.002	1.25	0.33	1.67
Farmer garden, sample 2 (Cluj county, Apahida, Corpadea village), sample 6	4.20	1.43	0.28	0.008	0.002	1.44	0.48	1.96
Supermarket, sample 7	5.50	1.38	0.25	0.012	ND *	1.95	0.24	1.81
Commission Regulation (EC) No 1881/2006 of 19 December 2006 and Commission Regulation (EU) 2015/1005 amending Regulation (EC) No 1881/2006 as regards maximum levels of lead in certain foodstuffs	-	-	-	0.2 **	0.05	-	-	-
FAO & WHO [54]				0.1 ***				

* ND—not detected. ** Authors assimilated sea buckthorn berries with “Cranberries, currants, elderberries and strawberry tree fruit” (Subsection 3.1.17 of Commission Regulation (EU) 2015/1005), as there are no specific values for sea buckthorn berries. *** For the category “Berries and other small fruits”.

**Table 4:** Results of binary logistic regression analysis.

Independent Variable	Dependent Variable	B	S.E.	Wald	df	*p*	OR
Wild SBT: Perceived risk of contamination with toxic residues	SBT consumption	0.004	0.005	0.580	1	0.446	1.004
Cultivated SBT: Perceived risk of contamination with toxic residues		−0.003	0.005	0.396	1	0.529	0.997
Perceived effect of SBT consumption on respondent’s health		−0.517	0.076	46.786	1	0.000	0.597
Average concern about various risks related to risk of SBT consumption		0.243	0.384	0.399	1	0.528	1.274
Constant		2.585	0.911	8.049	1	0.005	13.259

B: Regression coefficient; S.E.: Standard error; Wald: Wald statistic; df: Degrees-of-freedom; *p*: Significance; OR: Odds ratio.

**Table 5:** Results of Kruskal Wallis test with the post-hoc test and Bonferroni correction for H3a.

Pairwise Comparisons of Preferences for a Certain Type of SBT Products					
Sample 1–Sample 2	Test Statistic	Std. Error	Std. Test Statistic	Sig.	Adj. Sig. ^a^
Wild-Cultivated	−38.289	13.342	−2.870	0.004	0.012
Wild-Indifferent	−42.128	14.184	−2.970	0.003	0.009
Cultivated-Indifferent	−3.839	14.621	−0.263	0.793	1.000

^a^ Significance values have been adjusted by Bonferroni correction for multiple tests. Each row tests the null hypothesis that the Sample 1 and Sample 2 distributions are the same. Asymptotic significances (2-sided tests) are displayed. The significance level is 0.05.

**Table 6:** Research hypotheses proposed in the study and the results associated with them.

Hypothesis	Results
**H1**. *The SBT from spontaneous (wild) flora contains more toxic residues compared to cultivated SBT*.	H1 was confirmed.
**H2**. *People perceive a higher risk of contamination for wild SBT compared to cultivated SBT*.	H2 was confirmed.
**H3**. *There is a difference among people who prefer SBT from wild sources, those who prefer cultivated, and those with no preference regarding the perceived risk of wild (H3a) and cultivated (H3b) SBT contamination with toxic residues because they grow on contaminated land*.	H3a (focused on wild SBT) was partially confirmed. Differences regarding the contamination risk of SBT from wild origin were observed between: - people who preferred wild SBT and those who preferred cultivated SBT; - people who preferred wild SBT and those with no preference No difference was found between people who preferred cultivated SBT and those with no preference. H3b (focused on cultivated SBT) was rejected.
**H4**. *SBT consumption is predicted by (i) the perceived risk of contamination with toxic residues of wild SBT, (ii) the perceived risk of contamination with toxic residues of cultivated SBT, (iii) the average level of concern about various risks related to SBT, and (iv) the perceived effect of SBT consumption on respondent’s health.*	H4 was partially confirmed. The variable “perceived effect of SBT consumption on respondent’s health” has prediction power on SBT consumption, while the other three tested variables do not have contribute significantly to the prediction of SBT consumption.

## Data Availability

The data that support the findings of this study are available from the corresponding author on reasonable request.

## Appendix A

**Table A1:** Investigated variables, the questions used for each variable in the questionnaire for each group (SBT consumers and non-consumers), and the answer options presented to respondents.

Investigated Variable	Respondent Group *	Question	Answer Option
1. Consumption of SBT	SBTc/ SBTnc	Do you consume SBT?	1 = Yes 2 = No
2. The frequency of SBT consumption	SBTc	Some people eat sea buckthorn daily, others several times a week or a month, and others do one or more treatments a year, with a duration preferred by each. Estimate how many days a year you consume SBT products?	Number of days/year:….
	SBTnc	Why do you not consume SBT?	(a) I do not have time (b) I do not care (c) I don’t like the taste (d) I don’t like the smell (e) SBT do not do me any good (I am allergic to it, it causes me acidity, etc.) (f) It seems complicated to me to consume it (g) I think it doesn’t have a significant beneficial effect on my health (h) I do not trust the quality of SBT available on the market (i) Another reason (I do not know its properties; it was not recommended by doctor; I use other plants) (multiple answers can be selected)
3. Preference for SBT products from spontaneous flora against SBT from cultivated plants	SBTc	What do you prefer to buy when you have a choice?	1 = SBT with ingredients from spontaneous flora 2 = SBT with ingredients from cultivated plants 3 = Indifferent
	SBTnc	If you were to consume SBT, what would you prefer to buy, when you had the choice?	
4. Importance of wild origin of SBT products	SBTc	How important is for you that the SBT products you consume are from spontaneous flora?	Write a percentage that indicates importance for you on a scale from 0 to 100, where, for example: 0 = you are not interested at all ……., 50 = you are interested in the average measure, ………. 100 = you are extremely interested
	SBTnc	If you consumed SBT, how important would it be for you that the SBT you eat are from wild plants?	
5a. Perception of the risk level of contamination with toxic residues of SBT from wild plants because they grew on contaminated mining land. Refer to SBT products obtained from wild plants	SBTc	How high do you think the risk is that the SBT you consume and that were obtained from wild plants contain toxic residues because the plants grew on land with mining residues?	Write a percentage on a scale from 100% to 0%: ………. Meaning: 100% = sure they contain toxic substances due to the fact that the plants grew on land with mining residues; 75% = there is a 75% risk of containing toxic substances due to the fact that the plants grew on land with mining residues; 50% = I don’t know: maybe yes, maybe no; 0% = certainly they do not contain toxic substances due to the fact that the plants grew on land with mining residues.
	SBTnc	If you consumed SBT from wild plants, how much do you think there would be a risk that they would contain toxic substances due to the fact that the plants grew on land with mining residues?	
5b. Perception of the risk level of contamination with toxic residues of SBT because they were cultivated on contaminated mining land. Refer to SBT products obtained from cultivated plants	SBTc	How high do you think the risk is that the SBT you consume and that were obtained from cultivated plants contain toxic residues because they were cultivated on land with mining residues?	Write a percentage on a scale from 100% to 0%: Meaning: 100% = sure they contain toxic substances due to the fact that the plants were cultivated on land with mining residues; 75% = there is a 75% risk of containing toxic substances due to the fact that the plants were cultivated on land with mining residues; 50% = I don’t know: maybe yes, maybe no; 0% = certainly they do not contain toxic substances due to the fact that the plants were cultivated on land with mining residues.
	SBTnc	If you consumed SBT from cultivated plants, how much do you think there would be a risk that they would contain toxic substances due to the fact that the plants were cultivated on land with mining residues?	
6. The perceived effects of SBT consumption on the health	SBTc	How do you consider the effects of SBT products	0 = Very harmful…5= no effect…10 = Very beneficial
	SBTnc	If you consumed SBT, how do you think their effects would be on your health?	
7. Concerns about various risks of SBT-based products	SBTc	Are you worried about the following risks related to the SBT products you consume? (a) They are counterfeit (b) They are inferior in quality (they contain poor quality ingredients, e.g., the plants were not harvested at the right time) (c) They do not comply with hygiene standards (d) They contain pesticides (e) They contain hormones (f) They contain drug residues (g) They contain additives (h) They contain genetically modified organisms (i) They contain other toxic residues because they grew on soil contaminated with mining residues (j) The origin of the ingredients is unknown (k) They also contain other ingredients which effects I do not know, and this can affect my health (l) When SBT products are taken with certain medicines prescribed by a doctor, the effects can be dangerous (for example, the energizing action of SBT may explain the state of hyperexcitability; the blood thinning action of SBT may explain, for example, nasal bleeding, so, caution is recommended when administering SBT with Clopidogrel ratiopharm / Plavix). (m) They will not produce the desired curative effects (n) They can create addiction (o) They have not been controlled / tested by the manufacturing companies to verify if they are safe for the consumer’s health (e.g., they do not contain toxic substances, other substances harmful to health, etc.)	
	SBTnc	If you consumed SBT, would you be concerned about the following dangers associated with these products? **	
	SBTc	What else worries you about your consumption of SBT?	Open answer
	SBTnc	What else would you worry if you consumed SBT?	

* SBTc: SBT consumers; SBTnc: SBT non-consumers. ** The same options [(a) to (o)] were tested for SBTnc as for the SBTc.
